# Giant Papillary Renal Cell Carcinoma: A Rare Case of a 31-cm Renal Mass With Suspected Metastatic Disease on Imaging

**DOI:** 10.7759/cureus.117449

**Published:** 2026-10-04

**Authors:** Maram Aljuaid, Razan Alotaibi, Waad Abu Nakha

**Affiliations:** 1 Department of Radiology, King Khalid University Hospital, Riyadh, SAU; 2 College of Medicine, King Saud University, Riyadh, SAU

**Keywords:** atypical renal tumor, metastatic renal cell carcinoma, mri, papillary renal cell carcinoma, renal cell carcinoma

## Abstract

Papillary renal cell carcinoma (pRCC) is a recognized subtype of renal cell carcinoma (RCC) with variable clinical and radiologic presentations. Exceptionally large tumors with metastatic involvement represent an unusual presentation. We report a case of a massive pRCC with metastatic disease, highlighting its aggressive clinical and radiologic features. A 50-year-old male presented with a right flank mass and abdominal pain persisting for several months. He had no history of smoking or known genetic syndromes. Laboratory investigations showed mild anemia and microscopic hematuria, while renal and liver function tests were within normal limits. Imaging studies, including computed tomography and magnetic resonance imaging, revealed a large centrally necrotic right renal mass measuring 24×19×31 cm, with associated lung and bone findings, concerning for metastases. A renal biopsy confirmed the diagnosis of papillary renal cell carcinoma.

This case highlights an atypical presentation of pRCC, with an exceptionally large tumor size, T2 hyperintensity, central necrosis, and radiologically suspected metastatic disease at the time of diagnosis. While pRCC is generally smaller, weakly enhancing, T2 hypointense, and less aggressive than clear cell RCC, this case demonstrates that exceptionally large renal masses may have atypical imaging appearances. Therefore, pRCC should remain within the differential diagnosis, and histopathological confirmation is required for definitive diagnosis. Management of metastatic pRCC should be individualized based on disease burden, resectability, patient factors, and available systemic treatment options. Further research is warranted to better understand the biological mechanisms underlying aggressive tumor behavior. This report aimed to highlight a rare case, emphasizing its clinical significance due to its unusual presentation, notable mass effect, complexities in differential diagnosis, and radiologically suspected metastatic involvement. Specifically, we describe a case involving a sizable, necrotic, and contrast-enhanced pRCC that deviates from the conventional clinical and radiological features commonly associated with this tumor subtype.

## Introduction

Background on renal cell carcinoma (RCC)

Brief Overview of RCC Types and Prevalence

Renal cancer is ranked as the 14th most common cancer globally, with over 434,840 new cases and 155,953 deaths reported in 2022 [1]. Renal cell carcinomas (RCCs) represent a diverse group of tumors that originate from the nephron and are the most frequently diagnosed type of kidney cancer worldwide [2], with an average age at diagnosis around 57 years and a notable male predominance (approximately 60%) [3]. Internationally, the main histological subtypes of RCC include clear cell renal cell carcinoma (ccRCC) (75-85%), papillary RCC (pRCC) (10-15%), and chromophobe RCC (5-10%), which together account for around 95% of RCC cases [4,5]. Renal medullary carcinoma (RMC) is highly rare, accounting for less than 0.5% of all renal carcinomas [6].

RCCs generally remain asymptomatic until advanced stages, with over 50% of tumors detected incidentally due to widespread imaging [7,8], contributing to a trend toward smaller tumor sizes and earlier stages [9]. Only about 10-15% of patients present with the "classic triad" of flank pain, hematuria, and flank fullness [10]. Early clinical manifestations of RCC may be absent or non-specific; however, flank discomfort, hematuria, anemia, weight loss, and other constitutional symptoms may occur and should prompt appropriate clinical evaluation and imaging when indicated [10].

Papillary renal cell carcinoma (pRCC) features

Grossly, pRCC is well-defined, with a thick fibrous capsule, and is grayish-white to yellow and soft. On the cut section, it might show a papillary or cystic appearance with fibrovascular stalks and hemosiderin deposits [11]. According to the 2022 World Health Organization classification of urinary and male genital tumors, routine subdivision of pRCC into the historical type 1 and type 2 categories is no longer recommended, as the former type 2 category comprises a heterogeneous group of morphologically and molecularly distinct tumors [12].

Typical Imaging Presentation

Finding and staging the main tumor are the objectives of radiologic imaging. For the majority of institutions, contrast-enhanced computed tomography (CT) is the primary imaging method used to assess the intra-abdominal component of kidney malignancies and distant metastasis [13]. In general, pRCC is known for presenting as small, well-defined, homogeneous, hypovascular, and uncalcified tumors. The radiological findings of pRCC commonly include small size (usually <7 cm), lack of significant enhancement on CT, homogeneous texture, and absence of calcifications. A study revealed that pRCC typically appears hypointense on T2-weighted images in 89% of cases, specifically for tumors under 3 cm in size; the mean enhancement percentage is 68-69% [14]. It is known that the most common sites of metastases for RCCs are the lungs, brain, liver, and lymph nodes [15]. Furthermore, one study found a median tumor size of 3.9 cm in patients with localized RCC and 8.1 cm in patients with metastatic RCC [16].

RCC can present with uncommon radiologic features that make diagnosis challenging. These atypical manifestations include small RCC with concurrent adrenal metastases and RCC associated with significant abdominal lymphoma [17,18]. Other uncommon presentations include multiseptated cystic carcinomas that mimic moderately complex renal cysts on imaging, para-aortic lymphadenopathy as the only indicator of an undetected primary renal tumor, RCC resembling angiomyolipomas, and RCC occurring in the setting of acquired cystic kidney disease due to hemodialysis or hereditary polycystic kidney disease [18].

Why Early Detection Is Often Missed

A major diagnostic difficulty stems from the resemblance between pRCC and hyperdense cysts, making it challenging to distinguish benign from malignant lesions. This issue is especially evident in small tumors (less than 3 cm) or those exhibiting limited intra-tumoral vascularization, where CT imaging frequently misidentifies pRCC as hyperdense cysts, increasing the risk of misdiagnosis. Additionally, the diagnostic process becomes even more complex when tumors display cystic or necrotic characteristics, complicating the differentiation between pRCC and clear cell carcinoma [19].

## Case presentation

A 50-year-old male with a known history of type 2 diabetes mellitus presented with a right flank mass, dull abdominal pain, and lower extremity edema for several months. He was a non-smoker and had no known history of genetic syndromes. The patient was clinically stable with no recorded hypertension. On physical examination, the patient had a large, firm, palpable right flank mass measuring more than 20 cm, with bilateral lower-limb pitting edema (right more than left). Laboratory tests showed that the patient was mildly anemic (hemoglobin: 11.3 g/dL). Renal and liver function tests were within normal limits. No electrolyte abnormalities were noted. Urinalysis showed microscopic hematuria.

Imaging

Contrast-enhanced CT of the abdomen was performed and demonstrated that the right kidney was displaced inferiorly into the right hemipelvis. A large, heterogeneous, peripherally enhancing necrotic mass arose from the right kidney, displacing abdominal organs and major vasculature to the left hemiabdomen. The mass displaced the right hemidiaphragm and liver superiorly without definitive signs of invasion on imaging. Despite the significant mass effect on the adjacent major vessels, no evidence of venous invasion or tumor thrombus was identified. Abnormal enhancement of the thoracic vertebral body was noted, suspicious for osseous metastatic disease. No abdominal or pelvic lymphadenopathy was identified. CT chest demonstrated bilateral lower lobe-predominant lung nodules concerning for metastasis (Figures 1a-1c).

**Figure 1:**
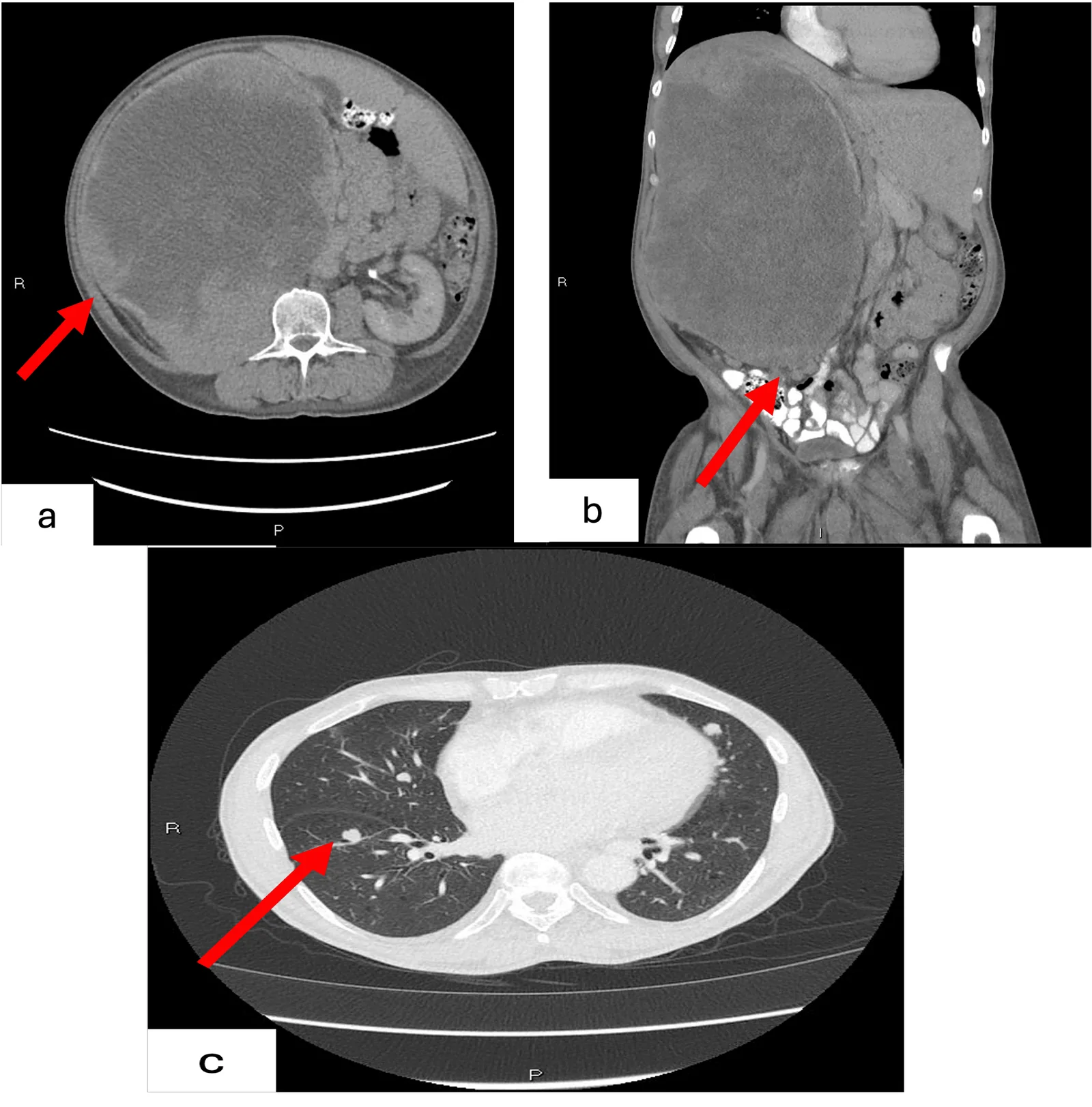
Contrast-enhanced CT in the portal venous phase. (a, b) Contrast-enhanced CT in the portal venous phase demonstrated that the right kidney was displaced inferiorly and anteriorly into the right hemipelvis. There was a large exophytic, centrally necrotic mass measuring 24×19×31 cm, with peripheral nodular enhancing soft tissue that was more prominent in the posterior aspect of the mass. The mass exerted mass effect, causing contralateral displacement of the abdominal organs to the left and elevation of the liver and right hemidiaphragm. No venous thrombus or abdominal lymphadenopathy was noted. The arrow in (a) indicates a large exophytic renal mass, while the arrow in (b) indicates an inferiorly and anteriorly displaced right kidney. (c) CT of the chest in the lung window demonstrated bilateral scattered pulmonary nodules concerning for metastases. The arrow in (c) indicates a representative pulmonary nodule concerning for metastatic disease.

Contrast-enhanced MRI of abdomen and pelvis was performed for further evaluation. The right kidney was displaced to the right lower pelvis. Arising from the right kidney was a 24 x 19 x 31 cm (anteroposterior, transverse, and craniocaudal dimensions, respectively) centrally necrotic, T2 heterogeneous, enhancing mass with multiple progressively enhancing peripheral nodular soft-tissue lesions, most prominent along the posterolateral aspect. The lesion exhibits mass effect on the right hepatic lobe without evidence of direct invasion. There is loss of the fat plane between the posterior tumor and right psoas muscle, with adjacent enhancing soft-tissue nodularity, concerning for invasion. The renal artery is patent. No definite tumor thrombus is seen. Abnormal enhancement of the thoracic vertebral body was noted, suspicious for osseous metastatic disease (Figures 2a-2g).

**Figure 2:**
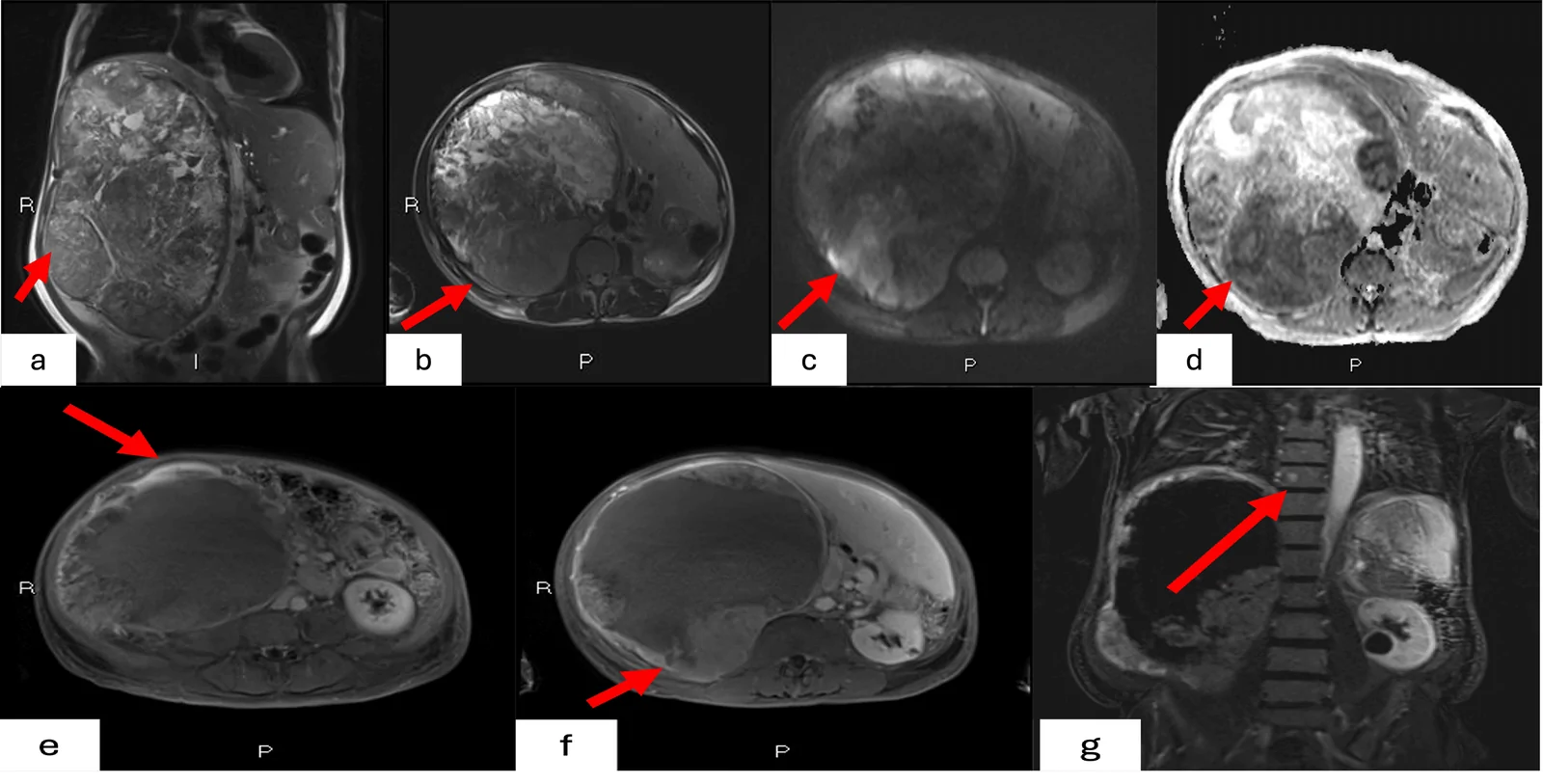
Contrast-enhanced abdominopelvic MRI. (a, b) Coronal and axial T2-weighted images demonstrated a large heterogeneous right renal mass (arrows). (c, d) Diffusion-weighted imaging (DWI) and apparent diffusion coefficient (ADC) images demonstrated diffusion restriction within the solid peripheral component of the mass (arrows). (e, f) Post-contrast T1-weighted fat-saturated images showed anterior and inferior displacement of the right kidney, with a large peripherally enhancing mass arising from its posterior aspect (claw sign). The mass exerted mass effect on the IVC, which remained patent, and abutted the psoas muscle without definitive evidence of invasion. The arrow in (e) indicates the displaced right kidney, while the arrow in (f) indicates the enhancing solid peripheral component of the mass. (g) Coronal post-contrast subtraction T1-weighted image demonstrated abnormal enhancement of a thoracic vertebral body (arrow), suspicious for osseous metastatic disease. IVC: inferior vena cava

Based on the imaging findings, the differential diagnosis for this large necrotic renal mass included clear cell renal cell carcinoma (ccRCC), chromophobe RCC, medullary RCC, and mesenchymal renal tumors such as primary renal Ewing sarcoma and leiomyosarcoma. The patient subsequently underwent nephrectomy, with surgical pathology confirming papillary renal cell carcinoma (pRCC) (Figures 3a, 3b). The available pathology documentation did not include a WHO/International Society of Urological Pathology (ISUP) grade. He subsequently continued systemic treatment at an outside hospital, so details of the specific treatment regimen, response, and subsequent clinical outcome were unavailable in our records.

**Figure 3:**
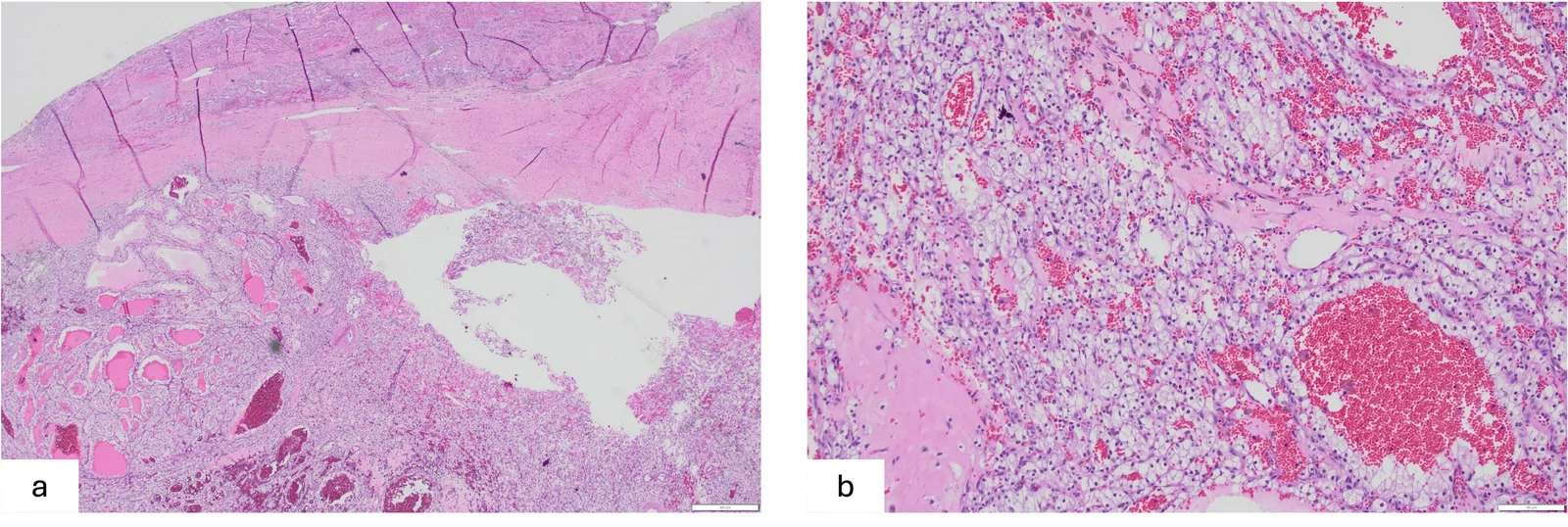
Histopathological appearance of the pRCC under the microscope. Sections show a malignant neoplasm composed of nests, alveoli, and cords of polygonal cells surrounded by a delicate, branching vasculature ("chicken-wire" appearance). The tumor cells have clear to granular cytoplasm due to abundant intra-cytoplasmic glycogen and lipid, and round to oval nuclei. The stroma is mainly composed of hemorrhage and cystic changes. (a) Original magnification 40× and (b) original magnification 200×. pRCC: papillary renal cell carcinoma

## Discussion

Comparison to previously reported cases

Massive pRCCs exceeding 30 cm are exceedingly rare, with very few cases documented in the literature, as most pRCCs are detected at significantly smaller sizes, often incidentally during imaging for unrelated conditions. This case highlights an atypical presentation of pRCC, in terms of tumor size, central necrosis, radiologically suspected metastatic disease, and high T2 SI, measuring 24×19×31 cm (anteroposterior, transverse, and craniocaudal dimensions, respectively). The dimensions observed in this patient markedly exceed typical expectations, as most reported pRCCs present at substantially smaller sizes [20]. Such unusually large pRCCs are rare, as evidenced by existing literature. For instance, a 43 cm, 13.0 kg pRCC in a 59-year-old male was characterized by extensive necrosis and a fibrous capsule [21]. Similarly, a 75-year-old male underwent resection of a 28×25×15 cm encapsulated pRCC, necessitating complex surgical intervention with favorable outcomes [22]. Another study further underscores this variability, describing an 18 cm polycystic pRCC [23]. Together, these exceptional cases illustrate the broad morphological spectrum of pRCC and challenge conventional size-based diagnostic assumptions.

Clinical and imaging presentation

RCCs generally remain asymptomatic until advanced stages, with over 50% of tumors detected incidentally due to widespread use of imaging [7,8]. The clinical presentation of RCC varies depending on tumor size and extent at the time of diagnosis. Only a small percentage of patients, around 10-15%, present with the classic triad of flank pain, hematuria, and a palpable flank mass [10]. Similarly, patients with pRCC may also experience weight loss, flank pain, a palpable mass, hematuria, fatigue, fever, or night sweats, although most cases remain asymptomatic in the early stages [24]. In the present case, the patient presented to the hospital with a flank mass and edema. Such symptoms are common in large tumors that compress surrounding structures, including the renal capsule and nearby tissues. The lower extremity edema was likely related to the tumor’s mass effect on the adjacent structures; however, no venous occlusion was identified.

Differential diagnosis of large renal masses

The evaluation of a large renal mass requires careful consideration of a broad range of differential diagnoses, including both malignant and benign conditions. pRCC and ccRCC are among the most common malignant tumors that present as renal masses [10]. Given the atypical imaging appearance, the differential diagnosis extended beyond conventional RCC subtypes to include chromophobe and medullary RCC, as well as mesenchymal renal tumors such as Ewing sarcoma and leiomyosarcoma.

pRCC is usually well-defined and hypovascular, and may be multifocal or bilateral. In contrast, ccRCC often appears as a hypervascular mass with areas of necrosis and hemorrhage, reflecting its more aggressive nature. Sarcomatoid features can develop in either subtype and are typically associated with high-grade tumors and a poor prognosis due to their rapid progression. Mesenchymal tumors such as angiomyolipoma, renal sarcoma, or solitary fibrous tumors can resemble RCCs on imaging, but are distinguished by their histologic and immunohistochemical profiles. Accurate diagnosis depends on a combination of imaging features, clinical presentation, and pathological confirmation [10]. Final histopathology remains the gold standard for confirming the diagnosis of pRCC and guiding prognosis and further management.

Prognostic considerations

Overall five-year survival for pRCC has been reported in the literature to range between 61% and 78.6% [25,26]. High-risk features associated with poorer survival include distant metastasis, tumor size greater than 10 cm, older age, Black race, and undifferentiated grade IV cancers [25]. These factors remain the strongest predictors of outcome in pRCC under the current classification framework.

In the present case, the exceptionally large tumor size (>30 cm), imaging findings concerning for local invasion, and pulmonary nodules suspicious for metastatic disease reflect a high-risk presentation. Long-term survival will depend on the final histopathological subtype, completeness of resection, and vigilant follow-up for recurrence or metastasis.

Educational value and contribution to literature

This case highlights a rare, exceptionally large pRCC exceeding 30 cm, expanding understanding of tumor growth and progression. The case also addresses the diagnostic challenges presented by large renal masses, where pRCC can be mistaken for other types of renal tumors. Accurate diagnosis requires a multidisciplinary approach and emphasizes the importance of histopathological confirmation. Reporting such atypical cases enriches medical literature, enhances understanding of pRCC variability, and informs future diagnostic and management strategies for renal cell carcinoma.

## Conclusions

This case highlights an atypical presentation of pRCC, with an exceptionally large tumor size and radiologically suspected metastatic disease at diagnosis. While pRCC generally demonstrates different clinical and biological behavior compared with ccRCC, this case illustrates that it can present as a very large, extensively necrotic renal mass with atypical imaging features. pRCC should therefore remain within the differential diagnosis of large renal masses, with pathological confirmation required for definitive diagnosis. In the setting of metastatic disease, management should be individualized according to disease burden, resectability, patient factors, and available systemic treatment options. Further research is warranted to better understand the biological mechanisms underlying aggressive pRCC behavior.
